# A Survey of Recent Indoor Localization Scenarios and Methodologies

**DOI:** 10.3390/s21238086

**Published:** 2021-12-03

**Authors:** Tian Yang, Adnane Cabani, Houcine Chafouk

**Affiliations:** Normandie Université, UNIROUEN, ESIGELEC, IRSEEM, 76000 Rouen, France; Tian.Yang@esigelec.fr (T.Y.); chafouk@esigelec.fr (H.C.)

**Keywords:** trilateration, indoor localization, RSSI, machine learning, Kalman filter, fingerprint

## Abstract

Recently, various novel scenarios have been studied for indoor localization. The trilateration is known as a classic theoretical model of geometric-based indoor localization, with uniform RSSI data that can be transferred directly into distance ranges. Then, a trilateration solution can be algebraically acquired from theses ranges, in order to fix user’s actual location. However, the collected RSSI or other measurement data should be further processed and classified to lower the localization error rate, instead of using the raw data influenced by multi-path effect, multiple nonlinear interference and noises. In this survey, a large number of existing techniques are presented for different indoor network structures and channel conditions, divided as LOS (light-of-sight) and NLOS (non light-of-sight). Besides, the input measurement data such as RSSI (received signal strength indication), TDOA (time difference of arrival), DOA (distance of arrival), and RTT (round trip time) are studied towards different application scenarios. The key localization techniques like RSSI-based fingerprinting technique are presented using supervised machine learning methods, namely SVM (support vector machine), KNN (K nearest neighbors) and NN (neural network) methods, especially in an offline training phase. Other unsupervised methods as isolation forest, k-means, and expectation maximization methods are utilized to further improve the localization accuracy in online testing phase. For Bayesian filtering methods, apart from the basic linear Kalman filter (LKF) methods, nonlinear stochastic filters such as extended KF, cubature KF, unscented KF and particle filters are introduced. These nonlinear methods are more suitable for dynamic localization models. In addition to the localization accuracy, the other important performance features and evaluation aspects are presented in our paper: scalability, stability, reliability, and the complexity of proposed algorithms is compared in this survey. Our paper provides a comprehensive perspective to compare the existing techniques and related practical localization models, with the aim of improving localization accuracy and reducing the complexity of the system.

## 1. Introduction

Recently, the indoor localization problem has been a widely-discussed research topic, since indoor mobile robots have become popular for transporting and AI (artificial intelligence) communication services. Besides, the indoor localization is rather different from the outdoor one that classic GPS-assisted methods cannot reach the accuracy requirement of indoor positioning, let alone the indoor networks’ limited coverage range and the channel fading issues. The major indoor localization technologies [1,2,3] are: Wifi, bluetooth, Zigbee, UWB [4] (i.e., Ultra wideband), RFID [5] (i.e., Radio-identification), Ultrasound [6,7,8,9,10,11,12], iBeacons [13], etc. The indoor positioning can be divided into LOS and NLOS according to the deployment and the coverage range of the related APs. The analysis of localization issues in these different scenarios refers to different attenuation and channel models, which is depended on number, thickness and material of obstacles (e.g., the walls). To this regard, the collection of distance indication data should be considered with these attenuation factors in different fading channel scenarios.

RSSI is considered as the key indication data for indoor positioning, since the distance from the anchor points to the current position can be effectively estimated from RSSI, especially in a theoretical distance-based localization model such as the trilateration. However, since the RSSI measurement also suffers from diverse indoor interference, multi-path effect, noises, and the changeable channel conditions related to the dynamic environment, CSI (i.e., Channel State Information) has been taken into consideration in recent work [14], as a complement to RSSI for more precise indoor localization; also, some other measurement parameters, namely RTT, DOA (i.e., direction of arrival)/AOA (i.e., angle of arrival) [15], TDOA and TOA [16,17], are also utilized to improve the localization accuracy.

The indoor localization problem can be classified into two principal branches: machine learning methods [18,19,20,21,22,23,24,25,26,27,28,29] and filters-based methods [30,31,32,33,34,35,36,37]. Traditional supervised machine learning methods such as SVM (support vector machine), KNN (k-nearest neighbors), Naive Bayes, and decision tree methods are capable of solving the data extraction, matching, and notably classification issues on the localization problem. In addition, since the networks are evolving into more complex networks nowadays, more sophisticated supervised machine learning methods based on neural networks (NN) are proposed [18,21,28], such as ANN (artificial NN) [18], CNN (Convolutional NN) [28,38,39], DNN (deep NN) [21,28,29], RNN (recurrent NN) [38,40,41]. Most supervised machine learning methods concentrated on the training phase, which analyzes the mapping relationship between input and output layer through an unknown hidden layers’ set. The offline training phase allows us to update the parameters of channel states and realize a fast learning process that illustrates the inner logic relations of datasets, and this later contributes to build a reliable model for online testing [42]. Another recent trend of localization by machine learning methods is the application of fingerprint onto collected RSSI indicator data [24,43,44,45,46,47,48,49], or a virtual fingerprinting technique based on RSSI prediction [50] (i.e., RSSI radio map), instead of the traditional RSSI-based methods [51,52,53]. The trilateration method can provide a decent localization accuracy in static measurement scenarios, but it relies completely on the input RSSI data. In more complex network scenarios where the interference, reflection and refraction of signal forces are superimposed on the specific points, the fingerprint-based methods can outperform the trilateration methods in terms of localization accuracy, since the position estimation is based on the data matching algorithm with a set of reliable RSSI data selected from a pre-built fingerprint database. Compared to supervised learning, unsupervised methods [19,22] allow us to dynamically update the network weights or biases in a real-time online phase without spending too much time on the training phase. The unsupervised methods are more autonomous and dynamic so that no human interventions are needed to supervise the update of network parameters, that’s the reason why most unsupervised methods are designed for online testing phase.

For the filter-based methods, the particle filter (PF) [34,36,54] and Kalman Filter (KF) [30,31,32,33,55,56]-based methods provide a large number of localization solutions. These filter-based methods are generally consisted of three steps: Prediction, measurement, and assimilation. The major difference of particle and Kalman filter is that, Kalman filter is modeled by uni-modal Single Gaussian Model (SGM) with linear functions, whereas particle filter applies factored sampling techniques to indicate the probability density features and to realize dynamic tracking based on Gaussian Mixture Model (GMM). Besides, the extended Kalman filter (EKF) methods [30,32] are more suitable for indoor localization scenario that is non-linear system, e.g., multi-modal Gaussian Model, while PF methods apply Sequential Monte Carlo method to indicate the density function and to predict the current position, which is the reason that EKF is widely applied for accurate indoor localization. EKF is considered as an approximation of Gaussian distribution with non-linear functions, EKF must remove the second and higher-order derivative terms which contain during the linearization process, but still it is not suitable for strongly non-linear systems. Unscented Kalman filter (UKF) methods [31] can overcome the high complexity of EKF by replacing the Jacobian matrix and can effectively improve the localization accuracy with a sub-optimal solution. In addition, The Cubature Kalman Filter (CKF) methods [33] allow us to preserve completely the second-order state information. CKF is derivative-free method that approximates closely to the Bayesian filter, where the inherit linear properties and elements are adopted into the integrals’ computation (similar to linear KF) under the assumption that the non-linear function is given [57].

Existing surveys such as [1,2,3] focused too much on the fundamental wireless technologies themselves (e.g., RFID, Bluetooth, WLAN, etc.) and their physical characteristics to be applied for localization. Moreover, the classification of localization scenarios is not presented and the measurement techniques are not matched to the methods. An early work [58] first proposed to divide the indoor localization into LOS and NLOS scenarios for separate mathematical analysis. Nevertheless, systematic analysis of ML-based methods are still missing; the paper surveyed too much on mathematical basis of TOA instead of RSSI. In addition, the number of ML-based algorithms presented by this paper can hardly be convincing. A recent survey [59] indicated the advantage of applying ML: efficient information gathering. After introducing the motivations of using ML in indoor localization, the authors analyzed ML-based techniques following their own specifics, and divided them into different categories of research problems. But still, this survey ignored the comparison of performance metrics among these ML-based methods.

In this survey, as a key method for the indoor localization, the geometrical and algebraic principle of the trilateration is demonstrated in 2D and 3D situations. Then, a large number of recent machine learning (ML)-based and filters-based techniques are analyzed and classified. From the performance perspective, the localization accuracy, denoted by the error indication vectors (e.g., RMSE), is considered as the most significant positioning metric. Other important characteristics such as reliability, stability, robustness, and delay are analyzed through different existing methods. Lastly, the computational complexity is compared among these methods. In general, this survey gives a global overview of research hot-spot and future trends for the achievement of higher localization performance, which ranges from theoretic problem formulation, methods classification, to performance analysis and comparisons. The core indoor localization techniques such as fingerprinting-based methods are notably presented in this survey with practical use cases.

The major contribution of our paper is:

1. The trilateration model is presented as a general framework, which provides the geometrical and algebraic basis of indoor positioning system;
2. A large number of ML-based methods are introduced, ranging from data collection, data feature extraction, to data clustering and classification, notably the comparative analysis during offline/online training phases.
3. An interactive linking from measurement techniques to localization methodologies is built in this survey. The related localization methods are classified and are illustrated based on their application scenarios.
4. Taking into account the indicators defined by the ISO/IEC 10835 standard, the survey provides a comparative study of complexity, accuracy and other performance metrics, which reveals the advantages and drawbacks of the proposed algorithms.

The rest of paper is arranged as follows: in Section 1, the trilateration model is presented with examples; indoor localization scenarios and new research trends are presented by comparing and classifying the related methods in Section 2, for ML-based and filters-based methodologies, respectively; performance characteristics of these proposed methods are evaluated and compared in Section 3; the recent research trend and future topics are presented in Section 4; and finally, the paper is concluded in Section 5.

## 2. Localization: Mathematical Principle and Models

**Figure 1:**
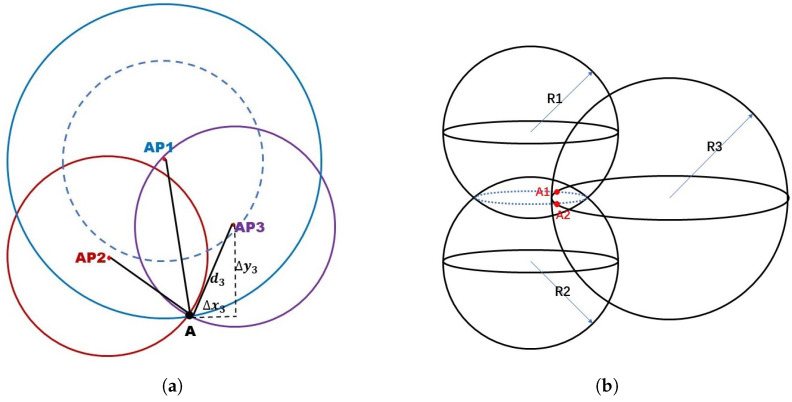
Trilateration models. (**a**) Ideal (solid line) model and practical error-tolerated (dash line) model for 2D trilateration. (**b**) Error-tolerated 3D trilateration model.

The trilateration localization problem is referred to the distance measurement and the position estimation process according to the geometric relations. The distance can be inferred from RSSI data based on the propagation model from anchor points (e.g., wifi access points) to the receptor. Then, by applying Pythagorean theorem, the measured distance can be expressed by the horizontal and vertical difference on x and y coordinates between the anchor points and the actual position, as presented in Figure 1a.

For three anchor points-based trilateration model, three geometry-based equations are formulated as a system of non-homogeneous linear equations for both 2D and 3D models [53]. For the ideal situation, the RSSI collected from each anchor point is considered as correct data, and only one intersection point A can be found as the solution of trilateration. However, taking into account the positioning error caused from inaccurate RSSI values, the circles might have a pair of intersection points as the dashed circle in Figure 1a, or even no intersection points if the positioning error expands. This is the reason why recent papers such as [22] showed great interest in improving the positioning accuracy in order to achieve the unique intersection point. Other work based on deep learning methods [28] also contributed to recover distance data and utilized the recovered output data for trilateration calculations. In the 3D model of trilateration, we also consider the practical model with positioning errors. Two circular arcs (denoted as blue dash line in Figure 1b) are considered as a set of possible solutions between two circles, and the third circle allows us to fix two intersection points (one single point in the ideal case), which corresponds to the two particular solutions of a system of non-homogeneous linear equations. Then, these two particular solutions form the general solution. In both 2D and 3D trilateration models, at least three anchor points coordinates and related RSSI data is needed in order to calculate the current position, according to the matrix formations as described in [53]. N > 3 anchor points case is also suitable for trilateration model, but the root-mean-square deviation (RMSD) should be seriously considered in this scenario, as the existing error variances of distance data might make it difficult to approach to a unique positioning solution. Several methods have been proposed to solve the positioning problem with RSSIs received from the anchor points. A classic solution [52,53] is to analyze RSSIs from the geometric relations. A conventional definition of RSSI to the distance d in the indoor localization problem is given as [60]:

(1) $$RSSI=-n*10*log_{10}\left(d\right)-A$$

where *n* is a propagation constant; A is the reference RSSI in dBm, which is depended on the defaulted channel attenuation in the space. More detailed calculations concerning the relational (i.e., between target and reference signals) expression of RSSI, and the RSSI definition applying intensity attenuation model are presented in [61,62], respectively. With the referential RSSI model, an extensive formula is given in [62,63] to define the RSSI at *d* distance to the transmitter:

(2) $$RSSI_{dB}\left(d\right)=RSSI_{dB}\left(d_{0}\right)-n*10*log_{10}\left(\frac{d}{d_{0}}\right)$$

where $d_{0}$ denotes the distance from referenced point to the common transmitter, and $RSSI_{dB}\left(d_{0}\right)$ is the corresponding RSSI measured at $d_{0}$ distance to the transmitter. *n* is the path loss exponent.

Authors of [53] formulated the 3D trilateration problem into a quadratic equation, two particular solutions are obtained, and these solutions are proved to be linearly independent so that a basis of Kern can be formed. However, RMSD (i.e., root-mean-square deviation) is not analyzed in [53]. In contrast, an error function is formulated in [52] as an objective function to be minimized, in a simplified 2D geometric model of the trilateration. B.Yang et al. applied Taylor expansion to prove that there exists a pair of coordinates near the selected target node, where the error range can be narrowed and the positioning accuracy can be improved by iterative process. Lastly, the authors utilized Bayesian filtering technique to estimate the posterior probability density function of target node in order to analyze the positioning accuracy. Apart from RSSI, the mathematical framework of measurement techniques such as DOA [64], TOA [65,66,67], TDOA [66], AOD, and AOA [68] is also formulated based on geometry analysis, and more details of these approaches are referred to in [64,68].

Even though the RSSI-based technique has very low complexity, the accuracy is too dependent on the dynamic indoor environment. The distance measurement of the RSSI-based technique is unpredictable, as it might suffer from severe signal attenuation and interference [69], as well as path-loss, fading, and shadowing. The simplicity of implementation is the main advantage for which the RSSI-based technique is widely applied for indoor localization. In contrast, ToA and TDoA-based measurement techniques can provide high positioning accuracy, with time synchronization the systems installed on both transmitter and receiver. Time synchronization results in high complexity of the system, but it contributes to higher reliability. The AOA-based measurement technique is proposed to provide faster real-time reaction along with higher accuracy compared to RSSI. Another advantage of the AOA-based technique is that it only requires two positions measuring equipment to locate the object [3], compared to at least three signal transmitters needed for RSSI-based localization. However, the accuracy of both ToA/TDoA and AOA is strongly dependent on the utilized signal bandwidth.

In addition, the study of trilateration has been extended into some particular localization systems such as ultrasound localization systems. A recent work [9] proposed to measure the distance between the anchor point and the users by multiple trilateration algorithms, according to the information collected from several monitoring ultrasound sensors, which are deployed in diverse indoor locations. The authors of [9] also pointed out one of the main disadvantages of ultrasound localization: the ultrasonic signal can hardly penetrate walls or other indoor obstructions. Moreover, the severe attenuation of the ultrasonic signal and the interference among its multiple reflections [8] can also deteriorate the system performance [16]. Therefore, ultrasound localization is not suitable for large indoor areas. Furthermore, costly and extensive hardware design [6,7] is required in order to overcome the inaccurate measurements caused by NLOS transmission and the multi-path effect of reflected signals. However, in a general case, the ultrasound system can provide decent real-time localization accuracy (i.e., normally in centimeters, but can be narrowed to less than millimeters by calibration steps [11]) notably towards moving indoor objects such as human beings, with an advantage of the quick set-up of ultrasonic sensors and the simplicity of its system structure. To improve the performance of ultrasound localization systems, several related works investigated the peaks of circular cross-correlation [7,8,10] function for TOF (i.e., time of flight) [7,8] and TOA (i.e., time of arrival) [10] measurements of ultrasonic signals.

Last but not the least, remarkable progress has been made over the last few years in the field of robotic indoor localization [70,71,72,73,74,75,76,77,78,79,80], especially towards the SLAM (i.e., Simultaneous Localization And Mapping)-related technologies [70,71,72,77,78,79,80]. SLAM allows us to locate the dynamic position of a moving object and to share the information of generated map with the other objects. SLAM is a highly-compatible technique with quick set-up and automatic maintenance, which provides a high flexibility using self-organized maps. However, the authors of [17] revealed that SLAM requires a large amount of processing resources to maintain the real-time localization service, and they also pointed out the design challenges towards its non-linear nature and error accumulation issue. J.Huang et al. [70] proposed to convert the signal strength-based SLAM into graph SLAM in order to address the posterior minimization problem by reducing to standard non-linear least squares. This approach allows us to further reduce the computational complexity comparing with the classic Gaussian process latent variable models (GP-LVM) [71]. The cooperative SLAM (C-SLAM) approaches [72,77] associated multiple mobile robots to simultaneously collect the localization information for map building, ensuring high localization accuracy prediction at an acceptable computational cost. Another robotic-related localization approach utilized the laser beams to activate the artificial landmarks, so that these landmarks can be detected and recognized by on-board RFID-readers [73]. Some other recent works surveyed visual SLAM [79], visual-LiDAR (i.e., Light Detection And Ranging) fusion based SLAM [78], as well as laser-visual fusion based SLAM [80]. Besides, the authors of [76] combined the 3D robotic SLAM model with the place recognizing technique using the training visual images (i.e., “visual words”), as well as other training data from multiple sensors such as WIFI signal strength, IMU (i.e., Inertial Measurement Unit), etc. Then, an empirical pedestrian motion model is applied to dynamically predict the robot’s location. In addition, the authors of [74] analyzed the robot’s TDOA measurement error model based on a pair of fixed UWB base stations. Then, they notably proposed an efficient measurement model, by performing close-form approximation, to simplify the previous model. Taking into account the adaptability to the NLOS path conditions, which varies over time, the proposed scheme in [74] took advantage of UWB localization, which provides extremely high localization accuracy during online estimation with the Expectation Maximization (EM) algorithm. A VLC-based (i.e., Visible Light Communication) robotic system [75] is proposed to improve the real-time robustness by applying the Bayesian localization model onto the Gaussian Process regression model, assisted by the light signal decomposition technique.

## 3. Localization Methods and Application Scenarios

Major existing methods for indoor positioning systems contain machine learning (ML)-based, filter-based methods, linear least squares (LS) methods [81], as well as the integrated framework of ML (e.g., general regression neural network) and filter-based methods [82]. In this section, the major interests of methodologies are ML-based and filter-based methods, to be analyzed with their measurement techniques and application scenarios, as in Section 3.1 and Section 3.2.

ML-based methods concentrate on the data manipulation, especially extracting and analyzing the useful location information via a data training process; filter-based methods lay emphasis on the current state switching, based on which a prediction of the next state is given accordingly. Then, the filter provides correction schemes to further improve the positioning accuracy and to lower the uncertainty of prediction. Besides, filter-based methods are more suitable for continuously changing systems.

### 3.1. Machine Learning (Ml)-Based Methods

Machine learning (ML) methods are composed of the offline training and validation/test process. An important percentage of collected data is used in the training phase in order to iteratively update and optimize the network parameters (e.g., weights and biases), whereas the remaining collected data is used to verify the trained system and are used for position predicting process.

Generally speaking, ML methods can be divided into supervised and unsupervised methods. Typical supervised learning methods are ANN (artificial neural network)-based methods [18], KNN (K-nearest neighbors) methods [24], decision tree [21] and support vector machine [21,29,83] methods, etc. Most of these methods applied classification or regression to recover or to refine the collected RSSI/distance data of trilateration. The classification process allows us to predict a discrete class label, while regression can be applied to predict the continuous quantity. The conventional unsupervised learning methods are based on clustering such as K-means methods [25], whereas the other unsupervised methods such as expectation maximization (EM) methods are proposed for unlabeled online scenarios. In addition, the unsupervised methods are more realistic than supervised ones, and the real-time trilateration solutions can be deployed without human interventions by unsupervised ML.

As an important branch of ML-based methods, neural networks (NN) are widely studied in the domain of indoor localization. Typical NN-based network architecture are ANN, CNN, RNN and DNN. ANN is known as the feed-forward NN that it works in a forward direction. The advantage of ANN is its great ability to deal with incomplete knowledge. In practice, an ANN-based back-propagation algorithm is usually applied to the training data collection [84], as it is robust against noise and interference. CNN is one of the most powerful NN-based learning methods, and it is more suitable for image-based data such as RSSI features image structure. Additionally, the CNN-based method is able to automatically detect the data features without human interventions. As the CNN-based method can capture spatial features from RSSI features images and can provide adequate recognizing accuracy, CNN is assumed to be an appropriate NN method for data features extraction, especially against the high dimensional input data. According to [27], RNN is normally applied to the data which has sequential correlation, for instance, the sequential RSSI measurement data [41]. The RNN-based method suffers from the vanishing gradient problem, and it is not capable of processing very long sequences. However, the RNN-based method contributes to provide precise prediction to the location [85], using its powerful sequence processing capability. It also has the nature of memorizing all the historical input information through time (even from a long interval of time steps, known as LSTM [27]). In addition, the RNN-based system possesses self-learning capability to correct the inaccurate predictions during the back-propagation process. the DNN-based model is widely analyzed in different localization scenarios [86,87,88], which notably focused on the robustness and reliability of features extraction for further location prediction and target classification using the DNN classifiers [86]. Another well-known advantage of the DNN-based method is that deeper and more complex functions are provided by DNN mapping the input to the output [87,88]. The authors of [87] pointed out one major weakness of DNN is the dependency of extremely large dataset while being trained during the offline phase. Thus, the DNN-based method is usually associated with other ML-based methods such as CNN and SVM for some complicated localization scenarios [28,29].

Based on a pair of RSSIs received from two reference positions, the Lambertian order is calculated in [18] so as to estimate the channel-modeling parameters (offline update); then, an ANN-based deep learning method is proposed to solve the trilateration problem for any unknown position (online position estimation). A suitable number of neurons is selected to match the number of hidden layers and the training performance. This method simplified the traditional algebraic-based trilateration solution and therefore the complexity is reduced, without lowering localization accuracy. However, the training progress and related ANN parameters should be suitable to the ANN structure, there is a lack of evidence that the proposed position estimator of [18] can always be self-adaptive and dynamic to any channel environment. Authors of [20] presented the importance of distinguish NLOS (non-line-of sight) from LOS (line-of-sight) conditions while analyzing RSSI samples. Two machine learning-based classifiers, namely support vector machine classifier and Gaussian Processes classifier, and a third hypothesis testing classifier are applied to identify the NLOS conditions, and three related regressors are defined for distance estimation. The method is robust, flexible, practical, and especially it has a high accuracy for both identification and distance measurement for both static and dynamic environments of NLOS. However, according to the authors, it is difficult to reduce the costly training phase, which is very dependent to the existing information such as the map and the training data of buildings. Another method based on iForest (unsupervised learning) [19] is proposed, integrated with supervised (e.g., K-nearest neighbors, random forest, etc.) and ensemble learning techniques [89]. The authors classified the data as normal/abnormal points according to the prediction of isolation forest, in order to improve the accuracy of estimation. Nevertheless, the improvement of position accuracy is rather limited compared to increased complexity brought by the proposed learning method.

Similar to visible light positioning [18,90], a recent work [21] also divided RSSI identification and classification process into offline and online stages for a device localization problem in wireless sensor networks. Since the moving object (e.g., a walking person) continuously shadows the wireless links built by several pairs of fixed WiLoc sticks and therefore affects the RSSIs, the two stages system allows to build classification model based on RSSI data, and dynamically predict the location based on interference detection influenced by his moving trajectory. The paper also applied data processing techniques to further improve the accuracy of the deep learning model. Still, this machine learning-based classification algorithm requires a high execution time, and the accuracy of localization is very reliable to the number of sensor nodes. A. Poulouse and D.S. Han [27] analyzed the performance of distance estimation in ultra-wide band (UWB) systems by correlating the received signals and reference signal at the reception of a UWB pulse (typically a Gaussian pulse) for indoor environment. Similar to the algebraic derivations of 2D trilateration problem as described in [52,53], the authors calculated the error term of non-homogeneous linear system, and they proved mathematically that there exists a pair of 2D coordinates to minimize this error term by applying MMSE (i.e., Minimum Mean Square Error) method. Furthermore, instead of using the RNN (i.e., recurrent neural networks) method, which suffers from the long-term dependencies and the vanishing gradient problem, they applied LSTM (i.e., long short-term memory) method to train the distance data collected from a specific trajectory, and update hyperparameter values by using regression model, until the best hyperparameter values are reached. Their simulation results show that the network loss ratio and localization accuracy outperforms other deep learning-based techniques, even considering different numbers of anchors. However, more specific trilateration scenarios should be considered other than a unique UWB pulse signal-based system with particular penetration and propagation characteristics. Therefore, the proposed LSTM method based on regression algorithm should be combined with the classification-based deep learning methods to maintain the positioning accuracy with other signal inputs and complex channel conditions.

**Figure 2:**
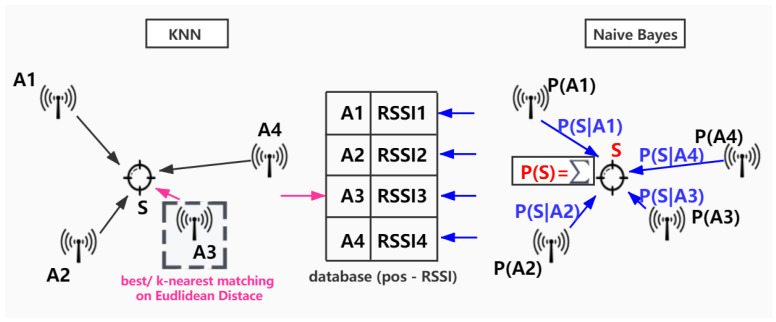
Comparison of fingerprinting KNN and Naive Bayes methods collecting RSSI data from existing database.

A recent work [24] contributed to analyze the performance of KNN and Naive Bayes methods (The structures of these two methods are compared in Figure 2), by comparing them with traditional trilateration method. The fingerprinting technique is especially utilized to build a database according to RSSIs measured at several points of interest, this step (i.e., the training phase) allows to improve the reliability of RSSI measurements. Then, the test data is sent to compare with stored values in RSSI database, in order to estimate the most-likely position, where RSSIs correspond accordingly to the closest value of database. To realize fingerprint-based localization, KNN is proposed to quantify the Euclidean distance between the received RSSI at specific location and the recorded fingerprint. The sorting and comparing process is iteratively executed until the k nearest matches are found. Besides, based on Bayes Theorem, the authors of [24] also proposed to estimate the receiver’s probable position according to all the mapped RSSIs’ records in the database, by calculating the probability of positioning decision with the sum of the products of RSSI measurement data appearance probability (in the database) and the transition probability of matching RSSI data to the estimated position. The authors compared the performance of KNN, Naive Bayes and trilateration in different scenarios under wireless technologies such as WIFI, bluetooth, Zigbee, bluetooth, UWB, RFID (i.e., Radio Identification), etc. They considered different densities of fingerprints, sizes of room and inference level as the variables of testbed settings. As a result, the trilateration method outperforms the fingerprint-based techniques in terms of computing complexity, with O(1) against O(mn) of KNN and Naive Bayes. However, the fingerprint methods have more accurate positioning results compared to the trilateration ones, KNN being the best at positioning accuracy among all, based on the precondition that the RSSI values could be acquired from a database that is already built, and at the cost of higher running time. Another fingerprinting technique is presented in [26], which proposed to apply deterministic and probabilistic techniques on a common test-bed, instead of a specific test-bed on the same paradigm. The authors of 14 proposed KNN-based method to overcome the shortage of the probability distribution-based methods such as Bayesian algorithms, the latter performs poorly with variable RSSI characteristics and multiple RSSI samples situations. Thanks to the standard deviation cut-off techniques, the method proposed in [26] allowed us to reduce the error distance and therefore improve the accuracy of indoor positioning with reduced dimension. In addition, a k-means-based clustering method is proposed in [25] to classify RSSI data. The authors proposed a RSSI partition algorithm based on the collected RSSI samples. The maximum, minimum and mean value of RSSI samples’ set are selected as the symbols of comparisons, then the distances between RSSI data and these three symbols are calculated accordingly and are compared with each other. The shortest distance among these three classes determines that the RSSI data belongs to the corresponding class. The proposed mechanism also allows us to improve online real-time performance by sample count and partition count: The IPS (i.e., indoor positioning system) is considered stable and real-time robust if at least 20 sample counts are detected; and the system is considered reliable if the tri-partition size count is subject to a normal distribution after applying the proposed filter. Thus, the accuracy, reliability and stability can be improved by the proposed method in a positioning testbed, especially comparing against the traditional methods such as the mean filter. Unlike [24], which completely replaced the trilateration by fingerprinting technique, the classification method proposed in [25] is still analyzed and tested on the traditional trilateration, the advantage of this method is simply the improvement on a sampling, clustering and filtering process with the raw RSSI data before working as the input of trilateration.

Instead of analyzing ML-based based methods with respect to the specific fingerprinting model, a recent work [91] evaluates the representations of inner characteristics of fingerprints in the high dimensional space. By applying 15 well-known public datasets, the authors of [91] applied quantitative correlation analysis for fingerprint pairs based on their normalization functions. The major objective of this research is to achieve an efficient reflection of spatial knowledge in the practical 2D/3D geometrical space by properly using the fingerprint data. This work also reveals a research tendency towards the generalization of fingerprint data analysis, independent of geometrical environment and data sources. Other recent contributions such as [92] focus on the correlation analysis among different fingerprints types. The authors indicates that only the highly-correlated data is admissible to construct the database so that the localization accuracy can be improved. Specifically, the proposed method in [92] selects RSSI and CSI as hybrid fingerprint data based on correlation coefficient, so as to construct a reliable fingerprint database, then deep learning approach is applied to further improve the accuracy. In addition, another work [93] provides a comprehensive comparison of existing clustering models in fingerprinting. The authors applied two aggregated normalized metrics as well as 16 heterogeneous datasets to evaluate the accuracy and computational cost of the clustering methods such as C-means clustering and Affinity Propagation Clustering (APC), with regard to conventional clustering methods as KNN and K-means. The major advantage of this work is the proposed evaluation framework containing different scenarios and metrics, which allows for comparative analysis. It is also pointed out in [93] that the computational cost of online stage relies on the number and the spatial distribution of APs and fingerprints, as well as the complexity on the reduction of radio map. By transferring the process of training data reduction into the offline pre-processing stage, sometimes the online computational burden can be mitigated, but the computational costs are still dependent on the implementation of clustering methods and notably their related evaluation scenarios (e.g., datasets).

A large number of references analyzed the clustering models [15,25,94,95,96,97,98,99,100] based on RSSI fingerprints data or radio map locations. The significant advantages of applying clustering models are the reduction of computational efforts and the improvement in localization accuracy [99]. From the technical perspective, clustering scenarios can be classified into stationary zone-based clustering and active motion-based clustering according to indoor activities. Besides, the cluster head selection rule and the definition of clusters’ number are drawing more research attention. In practice, KNN and K-means methods are considered as two classic clustering methods in this field. For instance, to cluster RSSI fingerprints, the supervised KNN method contributes to classify the current fingerprint according to the fingerprint data of K nearest neighbors around it, whereas the unsupervised K-means method groups the fingerprints which have common characteristics into the same cluster. Therefore, the K-means method allows us to classify the training dataset into *K* different subsets of similar fingerprints, and it can further improve the positioning accuracy. Even though the K-means clustering method can efficiently fix the optimized cluster, the authors of [96] revealed one of its shortages: the number of clusters is required in advance, and it lacks complete prior knowledge of the indoor area. In contrast, KNN has low complexity and it only requires low training time, providing a decent localization accuracy. However, it still takes quite a long time for the KNN method to execute an exhaustive search in order to determine the most-matched fingerprint from the fingerprint database. In addition to these two major methods, other extended clustering methods such as KWNN (i.e., K-Weighted Nearest Node) [95,99], fuzzy clustering [98,99], and soft clustering methods [100] are also proposed in the literature. More details about these methods and their application scenarios are presented in Table 1.

Some other articles contributed to recover the noisy distance data and to fill the missing information of EDM (euclidean distance matrix) [28]. This proposed deep learning-based method combined the classical completion and the neural network (NN) completion of EDM during a data preprocessing phase; more specifically, the authors applied the deep NN method to complete and denoise the distances by iteratively updating the network weights during the training phase, they also applied the convolutional NN to complete the distance and to further refine the completed data, as if the inter-nodes are subject to the communication conditions. The proposed deep learning scheme majorly focused on the trade-off problem between designing a generic offline model with high localization accuracy and decreasing online computational complexity. The authors highlighted the efforts on offline data completion and refinement phases by applying the different NN-based deep learning techniques. However, the system model of [28] is a simplified trilateration model under partial/full inter-nodes connection condition. More practical trilateration models should be considered and analyzed with proposed data completion schemes. Authors of a recent research [29] proposed class-independent LDA (i.e., linear discriminate analysis) techniques to reduce features from complex initial RSSI dataset without intervening information contents. Moreover, the proposed scheme is a hybrid scheme using both SVM and DNN to achieve relatively stable RMSE (i.e., root-mean-square deviation) performance in various indoor/outdoor scenarios. This proposed system combines the advantage of robust classification ability of SVM and the efficient prediction of DNN by using its deeper learning capability. The main contribution of [29] is that a scalable system is designed to suit different scenarios with reduced complexity and reasonable positioning error difference. The authors compared the mean error and the error distribution among those scenarios, and it turns out that RMSEs have limited differences and the tested positioning accuracy performance seems to be stable, but it still needs to be proved if the proposed scheme can outperform other NN-based learning techniques on positioning accuracy.

In addition, a unsupervised method is proposed [22] to apply NN-based techniques in a ranging module from RSSI/RTT (i.e., round trip time) measurements. The advantage of this module is that it can avoid the time-consuming collection of truth data (e.g., mobile devices’ coordinates) by system operators; instead, rich training data can be acquired from multiple devices using location services. Compared to [28], this method focused on the trilateration geometry in order to infer the accuracy of the proposed ranging module. Two cost functions are defined for both unsupervised and semi-supervised learning models, in order to achieve the intersection condition of 2D trilateration through the gradient computation. Moreover, the method proposed to compensate the characteristics of devices by iteratively optimizing the trainable variables, with a unique appropriate offset assigning to each device. However, the reliability and the complexity of this method still needs to be analyzed and compared to existing supervised learning methods. Other recent supervised and unsupervised methods are concluded in a survey [23] for different objectives. The application scenarios of several existing machine learning technologies are presented and classified, taking into account their advantages and drawbacks on performance metrics such as complexity, positioning accuracy, and delay. Detailed classification of aforementioned ML-based contributions are presented in Table 2.

Other contributions such as [38] work on the integration of NN-based techniques to avoid the limitation because of the non-Gaussian inverse problem. To realize the coordinates’ prediction with WIFI fingerprints, which is a sequential time-series regression problem, the authors of [38] proposed to add CNN in order to capture the features and to learn the Gaussian density of the complex high-dimensional input, before analyzing the state transition time-series data of hidden layers by RNN. This supervised location prediction methods is named as convolutional mixture density recurrent neural network (CMDRNN). Then, for further precising and recognizing accurate user location, semi-supervised methods based on variational autoencoders (VAE) are applied. VAE is a latent generative model where the latent variables of this model correspond to the continuous distribution of real user coordinates. The authors employed the deterministic and the stochastic predictor to calculate the user coordinates with learnt latent distribution from original input. This semi-supervised method is more stable than the MDN (mixture density network) on the learning process. This article combined the advantages of several new techniques to overcome the shortages of traditional networks, notably the neural networks. However, it remains questionable if the mixture of networks can realize adequate performance improvement with regard to the increasing computational cost. For example, the convolutional neural network layer is only applied to extract the features of high-dimensional input, CMDRNN is only a RNN-based method with the input data pre-procecssing module by a CNN-based layer, and this layer can be replaced by other MLE (maximum likelihood estimation)-based techniques than the proposed mixture density networks, which might occur computational instability and increase the system complexity. The major contribution of [38] is the introduction of VAE to realize the location recognition by unsupervised or semi-supervised learning procedure, which allows us to acquire the latent representations by learning from the unlabeled input data.

Last but not least, a new development trend has been revealed in recent papers [104,106], which analyze the effect of multi-path using machine-learning-based localization methods, especially related to NLOS identification and classification issue [101,102,103,104,105,106,107,108]. The authors of [104] studied the multi-path conditions between two transceivers, by analyzing the impact of obstacles on the prediction localization error. In this paper, three major ML classifiers: SVM, random tree and multi-layer perceptron, are applied with an experimental dataset in order to compare the distance errors among LOS, NLOS and multi-path conditions. As a result, the multi-path condition remains the hardest to be identified among all these three scenarios. Another work [106] compared the amplitude of multi-path NLOS signals to LOS signals in correlation function, taking into account the reflection and the diffraction of signals. The author applied SVM and NN methods for the classification to further observe the shapes of signal correlation outputs. Experimental results also revealed that the NN classifier slightly outperforms SVM on classification accuracy, at the cost of higher computational expenditure. Although the navigation satellite system is the proposed system in [106], an extension of this ML-based multi-path detection model to the scenario of indoor localization can be foreseen in the future work. Additionally, other recent works [45,50] contributed to the localization of multi-floor environment, especially with RSSI fingerprint database. G. Caso et al. [50] generated virtual RPs (i.e., Referred Points) based on RSSI data collection and data prediction through multi-floor propagation modeling. The authors applied deterministic Euclidean weighted KNN method to reinforce the positioning accuracy and to lower the prediction error. The paper highlighted the high feasibility of the proposed Vifi (i.e., Virtual Fingerprinting model) compared to other exhaustive RSSI data collection models and its contribution on multi-floor propagation analysis. However, this work didn’t mention other powerful ML-based methods such as SVM and NN, and a more persuasive conclusion on RSSI prediction accuracy still needs to be given with the performance comparison of other different methodologies. ELM (i.e., Extreme Learning Machine) and ensemble ELM methods are proposed by J. Yan et al. [50] to estimate floor-level and object position based on the fingerprint dataset. After the floor-level classification using a pre-processed training data subset, by generating and selecting the proper position regression functions, the authors achieved the minimum distance through refined localization process. More specifically, PCA (i.e., principal component analysis) is applied to reduce the dimension and to minimize the estimation error. In contrast to [50], more detailed performance analysis is presented by J. Yan et al. for multi-floor localization with/without PCA, especially the comparison with other existing ML-based methods such as k-means, KNN and SVM.

### 3.2. Filter-Based Methods

According to the structure, filter-based methods can be classified into finite memory structure method (FMS) [110,111] and recursive infinite memory structure (IMS) method (e.g., Kalman Filter). The classic nonlinear stochastic filters [112] is consisted of extended Kalman filter (e.g., [30,32]), particle filter, unscented Kalman filter (e.g., [31]) and cubature Kalman filter(e.g., [33]). The extended Kalman filter (EKF) is the most widely used method for indoor localization, however, unscented filter-based (UKF) method outperforms EKF method in terms of accuracy at a similar complexity level [34]. The Kalman filter methods are basically divided into two steps: the prediction and the measurement update.

Taking into account the uncertainty of process-noise covariance in the traditional extended Kalman filter (EKF) methods, J.M.Park [30] proposed a SEKFB (i.e., switching extended Kalman filter bank) method to roughly select a set of covariance hypothesis and operate them with the same number of EKFs in parallel, without using constant covariance values determined according to experience. State estimates are analyzed by Mahalanobis distance from the bank of EKFs, and the best estimate is selected as the output of SEKFB. The time difference of arrival (TDOA) is used to measure the indoor distance in [30], but this measurement suffers from noise, therefore KEF is applied with constant-velocity motion model to update and state the positioning variables. The proposed scheme can on the one hand lower the root time-averaged mean square error (RTAMSE), thus improving the positioning accuracy compared to other EKF methods; on the other hand, the reliability is also enhanced where less localization failures are detected for the SEKFB system. However, the proposed SEKFB scheme in [30] is still derived from EKFs, it is an application case of EKFs in a specific scenario with multiple process-noise covariance hypothesis. The base of algorithm design is similar with EKFs and the computational complexity hasn’t been improved.

A variational Bayesian-based method is proposed in [31] taking into account inaccurate process and measurement noise covariance matrices, especially considering the inaccurate and time-varying noise statistics. The authors applied unscented Kalman Filter instead of Jacobian matrix as used in traditional range-based methods (e.g., TOA, TDOA, DOA), so the complexity is reduced. The authors of [31] considered bounded noise on a RSSI-based system model, based on which the selection of sigma point set is defined. Then, the inverse Wishart (IW) distribution is applied as the conjugate prior distribution for the covariance matrix of a Gaussian distribution, notably with inaccurate state. By applying unscented Kalman filter, the proposed method allows us to execute time and measurement update for time-varying noise. An optimization algorithm is designed after the traditional steps of Kalman filters, that is, the initialization step, the prediction and update steps. This optimization algorithm is iteratively executed until the localization error is smaller than the threshold value. In this way, the localization accuracy and robustness are both ensured by applying proposed variational Bayesian adaptive unscented Kalman filter, with reduced complexity. This proposed method is purely a mathematical method inferring the likelihood and joint distributions for inaccurate state and noise covariance matrices. Somehow, the authors pointed out that the objective error function is determined in advance. Although this unscented Kalman filter method can effectively lower the complexity than the traditional range-based methods, more attention should be paid on deciding the lower threshold of error function, in order to avoid too strict threshold values, which causes too many repetitions using unscented Kalman Filter, and potentially increases the execution time.

Other than TDOA and RSSI-based methods as presented in [30,31], the authors of [32] applied Wifi Round trip time (RTT) as the ranging method. With a two-layer optimization-outlier detection scheme, an adaptive filter composed of multiple EKF is proposed for reducing localization error of wifi RTT, and at the same time lowering the packet loss rate and measurement jump rate of wifi RTT. A weighting scheme is added onto the position-tracking EKF’s measurement model and time/measurement update processes, in order to adaptively assign the suitable weights based on the total number of EKF built. The principle of outlier detection [113,114] is using the innovation and residual error optimization model so as to correct the state estimation, and to determine if a measurement is an outlier by comparing the residuals with the set threshold for each state estimation. Then, the pedestrian dead reckoning (PDR) [115] is proposed to improve the accuracy of wifi RTT, the authors applied a fusion-tracking federated filter (FF) to fuse the wifi RTT and PDR system based on observability, in order to further mitigate and correct the cumulative error inside the pure PDR system. Lastly, the authors proposed real-time fixed-interval smoothing method, adding a backward filtering technique to recheck the outputting results of fusion-tracking federated filter, and applying a weighting algorithm to integrate the results of backward and the fusion-tracking filters. To this end, the robustness of the proposed system is reinforced.

To analyze and optimize the attitude and heading estimation with readings of a Gyroscope, an accelerometer or a magnetometer, the authors of [33] applied quaternion-based adaptive cubature Kalman filter (CKF), which has a strong adaptability to correct the non-linear model without calculating Jacobian matrix. The fading memory weighted and limited memory weighted methods are applied to constraint the error and the measurement outlier. For the limited memory weighted method, the latest step data is applied as memory window in the pedestrian walking process. By applying these weighted schemes, the proposed adaptive CKF in [33] allows us to lower the weight of old data and to increase the weight of latest historical data. In this way, the accuracy of statistical properties estimations is improved. Moreover, the innovation sequence is utilized to reinforce to measurement of noise covariance matrices by analyzing and preventing the filtering divergence. Moreover, an adaptive factor based on predicted state discrepancy statistics is introduced to mitigate the sudden turns of abnormal state disturbance, in order to lower the large dynamic model errors of the filter and to correct the Kalman gain. Comapring to the Sage-Husa cubature Kalman filter (SHCKF) and other traditional CKF, EKF methods, the proposed adaptive CKF can improve the accuracy under quasistatic conditions, and eliminate inference from uncertain dynamic system noise. The simulation results show that the proposed method can also provide a more accurate and stable heading estimation performance with minimized mean absolute value of heading error, by analyzing the location tracking performance of PDR.

Other works such as [34,35,36] applied particle filter for indoor positioning. Instead of SBMP (i.e., sequence-based magnetic matching positioning), the authors of [34] proposed a SPMP (i.e., single-point-based magnetic matching positioning) method, which has significant advantage on the flexibility. In a Pedestrian Dead-Reckoning (PDR) module, after measuring heading angle by using the gyroscope, the corrected gyroscope data is formulated the summation of normalized gyroscope raw data and a function of gyroscope error correction item. Then, a finite-state machine (FTM) is employed to detect the walking steps, which are resistant to the interference and measurement errors, based on which the step detection is divided into five states against the interference of false peaks. Then, by transforming the three-axis geomagnetic features data to the geographic coordinate system (GCS) data, five geomagnetic features data are extracted as the fingerprint data. The classic PL-based on Monte Carlo method and the Bayesian theory has a decent performance on the prediction of optimal state estimation and appearance probabilities even against the complex integral operation, but still it suffers from the particle degradation problem with re-sampling process. To this end, the particle gene mutation algorithm is applied by mutating particles and reconstructing a particle set in order to improve the diversity and reliability of re-sampled particles. Finally, the fusion-positioning algorithm is designed to combine the PDR location with the geomagnetic position which further improve the accuracy and stability on positioning performance.

Another PDR-based scenario is presented in [35]. The authors utilized finite state machine (FSM) and decision tree (DT) methods to rapidly recognize and monitor the phone mode/motion by matching pre-defined threshold, which allowed them to efficiently detect the mode change without continuously extracting data features as in [34]. Thus, the proposed method is more timely and energy-efficient. DT is able to detect the current state of the user after FSM succeeds to detect the pose or mode change. The heading estimation (e.g., step-wise heading, global heading, and fusion heading) is realized by PF method and map watching technique, where global heading of pedestrian outperforms other headings such as step-wise heading in term of stability. The advantage of this study is that the realistic and real-time scenario applied for motion recognition, head estimation and localization, which allows a timely tracking and correction of trajectory. However, PF is only utilized as an assisted method for head estimation phase, more sophisticated filtering methods should be adopted to further improve the stability of system especially against sensor noise and measurement noise. In addition, Ref. [36] proposed to assign proportional weight to particle’s likelihood. The predicted and updated distributions are integrated, since the particle approximation of updated distribution relies only on previous updated distribution, instead of drawing particles on predicted distribution. This method avoids weighting wastes and unnecessary state transitions. The authors aim to achieve high accuracy with fewer particles, which remarkably improves the efficiency of particles’ use. However, the weighting phase is still a key technique, and it cannot be eliminated in the proposed algorithm. Moreover, the reliability is reduced with the integration of predicted and updated distributions. More work should be done to prove the feasibility of this method, and more attention should been paid to the improvement of approximation accuracy with a maximized weighting solution on the likelihood function. A comparison of filters-based methods are presented in Table 3.

## 4. Performance Evaluation Metrics and Indication Vectors

In 2016, ISO (i.e., International Organisation for Standardisation) and IEC (i.e., International Electro-technical Commission 18305) published a standardized document for methodologies of the test and the evaluation of indoor localization and tracking system (i.e., LTS), namely ISO/IEC 18305 [123]. According to [124], the performance metrics proposed by ISO/IEC 18305 correspond to several evaluation aspects such as the absolute positioning errors, the accumulated errors, as well as the velocity, the obstacle interference, the latency, the update rate, the coverage ratio, etc. The authors of [124] proposed a comprehensive scoring system to formulate the impacts of error vectors and other requirements of indoor localization system, by applying the evaluative indicators of ISO/IEC 18305 and allocating proper integration weights to these indicators.

- **Localization Accuracy** is the major target of performance evaluation. Based on the reception of RSSI or other measurement indicators (e.g., TDOA, TOA, AOA) at the current user’s position, the localization accuracy is influenced by the complex indoor environment, for instance the multi-path effects, the obstacles, the multiple interference and noises. More importantly, the accuracy is the primary evaluation task of algorithm performance. For this task, many contributions like [29] apply RMSE to reflect the localization accuracy. In the classic trilateration model, the accuracy can be guaranteed using mathematical methods such as algebraic methods. However, for more complex networks where multiple APs are detected, more erroneous information can be involved in algebraic-based calculation, thus the localization errors can be caused. That is the reason why the ML-based methods are introduced to refine and to complete the RSSI data before applying them into the trilateration process [28]. The authors of [28] also proposed the cumulative distribution function (CDF) as the integration of error rate PDF (i.e., probability density function) in order to quantify the localization accuracy, instead of directly measuring classic discrete error indicators such as RMSE. In addition, Ryosuke Ichikari et al. [124] proposed to utilize empirical CDF (eCDF), a discrete CDF function depending on the previously-collected samples, which allowed them to provide analytical statistics for absolute errors at different percentile levels (e.g., median-50th, 75th, 95th, etc.). The general accuracy of indoor localization is less than 0.5∼1 m, and the precision unity is per centimeter.
  Mathematical computations of CDF and RMSE are written as in (3) and (4), according to [28,29], respectively.
  (3) F(x)=P(error≤x)
  (4) $$RMSE=\sqrt{\frac{\sum_{i=1}^{n}{\left|{\hat{y}}_{{}_{i}}-y_{{}_{i}}\right|}^{2}}{n}}$$
  Another error measurement method MAE (i.e., mean absolute error) contributes to measure the arithmetic average of absolute error between the predicted value and the observed value. The expression of MAE is showed as follows:
  (5) $$MAE=\frac{\sum_{i=1}^{n}\left|{\hat{y}}_{i}-y_{i}\right|}{n}=\frac{\sum_{i=1}^{n}\left|e_{i}\right|}{n}$$
  where $|e_{i}\left|=\right|{\hat{y}}_{i}-y_{i}|$ defines the absolute error between the two vectors *y* and $\hat{y}$ through time.
  Apart from these conventional definitions of errors, other indicators defined by ISO/IEC 18305 [123,124,125] are also drawing high research interests, namely EAG (i.e., error accumulation gradient) and CE (i.e., circular error). The evaluation of EAG is known as the error accumulation speed notably related to PDR [125]. The median of CE is commonly applied as an absolute error indicator compared with the ground truth position under 2D model localization [125], representing horizontal error magnitude [126]. The ideal absolute position accuracy is up to 10 mm according to ISO/IEC 18305 [123,127]. In addition to CEP (i.e., circular error probable) based on horizontal 2D localization framework, other medians of spatial errors are presented as VEP (i.e., vertical EP) and SEP (i.e., spherical EP), corresponding to vertical and general 3D error magnitude, respectively [123,126], along with their related percentiles (e.g., CE95, VE95, SE95, etc.).
- **Stability** refers to the performance feature against the fluctuations in different scenarios especially against measurement noises. The localization system is supposed to remain stable even with incomplete or incorrect input data. In order to overcome the instability of traditional RSSI-based methods, a tri-partition RSSI classification method [25] is proposed and a RSSI filter is applied based on k-means clustering, in order to reduce the variance range for each sample and therefore improve the stability. This work has notably quantified the stability as sample standard deviation (SSD) ratio, calculated as the division of the standard deviation function to the mean function. For ML-based localization methods, slightly-changed training data should not affect or perturb the prediction results. A SVM-based ML method [29] evaluated the errors (i.e., RMSE, and the mean positioning errors in meters) in four different scenarios. The estimated error turned out to be less than 10 centimeters, which proved the stability of the proposed system. Other pre-mentioned techniques, such as VAE [38], also help to improve the stability of classic MDN, which suffers from unstable data during learning process, especially when the samples number is too large. For filters-based localization methods, the authors of [32] pointed out that the particle filters have poor stability on data fusion. They proposed to apply EKF-based data fusion method with RTT to eliminate the measurement errors and to keep the system stable. Moreover, PDR is applied to further resolve the packet loss of RTT in order to reinforce the high stability performance.
- **Reliability** is basically a performance feature towards real-time localization modeling, which requires the system to realize precise positioning [60] with acceptable roaming delay [59]. To reach the reliability requirement under real-time, the minimum sampling number for tri-partition-based RSSI filter [25] is set as 30 with the shortest RSSI collection time at less than 1 s. The minimum sampling number is deemed as acceptable as if the related tri-partitioned RSSI classification data is subject to normal distribution. Based on RSSI measurement, the authors of [128] applied reliability check algorithm onto nonlinear estimators namely the minimum variance (minVAR) estimator and the ML-based LSE (i.e., least squares) estimator. The results revealed that minVAR estimator can approach the reliability requirement by increasing the number of antennas adaptively, whereas RSSI measurement is perturbed and biased by measurement noises with LSE estimator. From these examples, we conclude that reliability refers to the capability to estimate unbiasedly the user’s position by collecting and analyzing RSSI data within acceptable latency. The design of positioning estimator also needs to be autonomous and adaptive in order to meet the system stability requirement.
- **Scalability** is defined as the capability to simultaneously support multiple devices under different user density. According to [129], the overall scalability of the UWB system is divided into PHY layer configurations, MAC schemes and localization approaches in order to reach the requirement of tag density. The authors of [129] investigated the combinations of three-layer schemes, and they found out one scalable solution is a random access approach (ALOHA) with short TDOA packages. As a conclusion of [129], the key factors which determine the scalability are the cell size (or maximum achievable range) and the user density. Other work such as [130] constructed a virtual radio map to guarantee the scalability in both surveyed and unsurveyed indoor areas. This proposed scheme can continuously improve the performance of localization by allowing user to upload their coordinates to the server. Another work [29] utilized the feed-forward neural network (FFNN) algorithm to fill the missed values of RSSI during online stage. Therefore, the system scalability can be guaranteed by continuous updates and automatic fills into the missed RSSI values when the trained AP cannot transmit WIFI signals.
- **Complexity** is the major computational concern of localization algorithm. Some existing works [39,130] utilized the RSSI heat maps or radio maps as an assisting techniques to reduce the system complexity. More importantly, the complexity should be considered on the algorithm design of all methods used in the localization system. Many studies [38] focused on highly-combined techniques to improve the system accuracy, however the complexity of entire system is also increased, as an addition of these multiple methods. The complexity is an important expense of localization algorithms and should be kept reasonable while analyzing other metrics such as accuracy. Otherwise, even if the accuracy is improved, the improvement on system performance is not convincing at the cost of increasing complexity. To this end, many contributions [18,28] proposed to reduce the complexity while building the networks, and the authors compared the complexity of their methods with existing methods to highlight the advantage. The complexities of representative techniques are listed in Table 4.

## 5. Research Trend and Challenges

Although ML-based methods are capable of extracting and classifying useful information for accurate localization, the efficiency of data processing is still dependent on the training data. For instance, neural networks may have a complex structure, and they also require a large amount of training data. Thus, how to determine the appropriate amount of training data for a specific ML method still remains a future task. In addition, existing supervised ML-based localization methods mainly focused on the training phases, whereas dynamic and efficient location estimation solutions are still lacking during the expensive and time-consuming online testing phase where human interventions are still necessary. For example, the extreme learning machine (ELM) has been widely applied as it offers a high localization accuracy. However, this technique requires quite a long testing time during the online phase. Therefore, it is more suitable for offline training phase and other methods such as WPL (i.e., weighted path loss), which are combined with ELM to provide faster estimation [131] during the online phase. Moreover, the standardization of available training data still needs to be addressed [23]. Another challenging task is the complexity reduction of the multi-dimensional data (e.g., data collected from fingerprinting radio map) and the storage saving, using ML-based dimension reduction techniques such as principal components analysis (PCA). Although these techniques have a decent performance, due to the huge volume of high-dimension, high-variety data, more powerful supervised and unsupervised methods should be developed to further reduce the computational complexity of data processing, considering the device heterogeneity and the dynamicity in the future networks.

The integration of localization systems with 5G infrastructure is also facing challenges, notably towards the critical designing metrics for localization devices such as low power consumption and low infrastructure cost. Although the application of 5G technologies is leading the localization systems to a single simplified infrastructure with low latency, cm-level horizontal accuracy, and floor-level vertical accuracy, the real-time localization still calls for energy-consuming tags and other heterogeneous devices. Moreover, to realize the real-time localization without any compromise on the positioning accuracy, it is necessary to further develop the cooperative positioning techniques, and to fuse the data from multiple sources of 5G devices (e.g., IMU, cameras, sensors, etc.) [132]. Other aspects such as the exploitation of NLOS and multi-path scenarios, the time synchronization, and the identification of application scenarios are drawing more and more research interests to 5G-oriented localization systems. Furthermore, the future technological topics such as intelligent reflective surfaces (IRSs), beamspace processing, integration of ML and AI (i.e., artificial intelligence), as well as more refined technologies such as THz band active/passive imaging, SLAM (i.e., Simultaneous localization and mapping), active/passive sensing framework, and channel charting have been raised and precised towards 6G localization in a recent work [133].

Following the standardized indicators and metrics defined in ISO/IEC 18305 [123], a recent survey [134] argued that the proposed test and evaluation (T & E) methods in ISO/IEC 18305 haven’t considered either the users’ features/the indoor elements, or more dynamic mobility modes other than the 5 basic motions (e.g., walking, sidestepping, etc.). Besides, the authors also pointed out that ISO/IEC 18305 only proposed T & E methodologies on a system level, instead of both system and component level as being previously defined, which cannot meet the developers’ requirement. In the future standardization, more T & E challenges need to be overcome: benchmark methodologies and dataset still need to be developed and designed; theoretical limit such Cramer–Rao lower bounds (CRLB) should be derived for the majority of localization scenarios; more evaluation strategies should be standardized for developers and testers, rather than for only end users [134].

Recent works contributed to the identification of LOS and NLOS phenomena, notably in the data features extraction phase. However, the NLOS identification and multi-path conditions remain important tasks to be studied, especially in the presence of moving indoor objects. Current research challenges such as multi-path delay profile (MDP) extraction, multi-path component analysis, and floor-level identifications are still fulfilled with more convincing methodologies. Another important tendency is the application of dead-reckoning (DR) techniques, which provide the current position prediction according to the previous states and positions. The advantage of DR is that it can provide an accurate and automatic localization using inertial sensors (e.g., accelerometers, gyroscopes, pressure, and magnetometer sensors) even the GPS technologies are temporarily unavailable [135]. However, DR technique is subject to cumulative errors and is therefore highly dependent on the walking distance of pedestrians, not to mention the sensor noises resulting in large drifts on estimations of user heading. To address these issues, some recent works proposed to apply VPR (i.e., Visual Place Recognition) technique, an image retrieval-based technique that assists to correct the cumulative errors of PDR and provides vertical floor-level recognition, instead of only 2D horizontal movement model by PDR [136]. In future works, regarding the unavoidable cumulative heading and step-length errors, more methods should be analyzed to compensate for these weaknesses of DR techniques, so as to realize continuous and long-term pedestrian localization. Last but not least, a novel tendency on the integration of multiple techniques is drawing more and more research interests, such as ensemble models mixing several neural networks, as well as the integration of NN-based methods with KF-based methods [82]. Although these methods have brought about the improvement in localization accuracy and reliability, the shortages of these integrated methods still need to be analyzed, notably against their structures of high complexity and high latency.

## 6. Conclusions

In this survey, various localization technologies are analyzed for indoor environment. Different measurement techniques (e.g., RSSI, TOA, TDOA, AOA, RTT, etc.) are presented, based on which a large number of machine learning-based and filters-based methods are proposed, so as to improve the localization performance metrics namely the accuracy, reliability, scalability, stability, robustness, etc. The complexities of algorithms are notably studied and compared. This survey follows closely the evolution of ML-based and filter-based methods, and it connects these existing methods to their application scenarios, data sources, and measurement objectives. This survey also draws attention to the implementation of typical techniques such as the RSSI fingerprinting techniques and NN-based classification/regression methods, as well as the identification of some complex indoor scenarios such as NLOS and multi-path conditions. By evaluating the ISO/IEC 18305 standard and several white papers towards the design of 5G and 6G-oriented localization systems, the survey focuses on the technological trends and research challenges towards the heterogeneous design and the future standardization of indoor localization systems.

## Figures and Tables

**Table 1:** Clustering method and the related clustering objects.

Reference	Clustering Object	Clustering Method
[25]	Tri-partition RSSIs	K-means
[94]	Location Fingerprints	Affinity Propagation Clustering (APC)
[95]	Wifi Fingerprints	K-means + KWNN (K-Weighted Nearest Node)
[96]	RSSI Radio Map	K-means + Mean-Shift
[97]	Zone-based RSSI data	K-means
[15]	RTT and AOA	Coordinates Clustering
[98]	Location Fingerprints	Fuzzy C-Means (FCM)
[99]	RSSI Fingerprint Map of 5G signals	KNN
[100]	Wifi Fingerprints	Gaussian Mixture Model (GMM)-based Soft Clustering

**Table 2:** Supervised/unsupervised machine learning methods.

Supervised ML	ANN based method in VLP (visible light positioning) [18]
	NLOS classification and mitigation based on RSSI [20] and TOA [58]
	DNN based device-free localization [21]
	CNN and DNN completion and refinement for EDM recovery [28]
	Hybrid SVM- and DNN-based method [28]
	KNN and Naive Bayes methods with RSSI fingerprints [24]
	CNN-LSTM-based hybrid deep learning with RSSI heat map [39]
	SVM and Gaussian Process regressions for LOS/NLOS identification, classification and error mitigation [101,102,103,104,105,106,107,108]
	ANN and CNN based method to identify and to estimate position of room with human object [109]
Unsupervised ML	Isloation forest-based classification method [19]
	Ranging module-based NN method for trilateration [22]
	k-means RSSI-based classification for improving accuracy [25]
	VAE-based semi-supervised learning model with latent variables [38]
	PDR-based reliable unsupervised approach with iBeacon corrections and fingerprint database auto-building [13]

**Table 3:** Comparison of existing non-linear stochastic filters methods and their objectives.

Ref.	Measurement Technique & Data Source	Filter/Method
[30]	TDOA	Switching EKF
[31]	RSSI	UKF
[32]	WiFi RTT	Adaptive EKF
[33]	Attitude & Heading	Adaptive CKF
[34]	Geomagnetic Multi-Features Data	Genetic PF
[36]	Target’s Cartesian Coordinates	Likelihood PF
[116]	RSSI, inertial sensors vectors, local map information	Rao-Blackwellized PF
[117]	IMU sensor data & Wifi RSSI fingerprints	LKF (Linear KF)
[118]	Inertial sensor data & Wifi radio map containing RSSI training pairs	EKF
[119]	Hybrid TDOA/AOA	EKF
[120]	DOA endoscopy capsule	UKF
[121]	TOA	EKF
[122]	TOF	discrete EKF

**Table 4:** Computational comparison of existing localization techniques.

Reference	Technique	Complexity	Symbol and Notation
[18]	ANN-based deep learning techniques	$O\left(N_{neurons}^{2}\right)$	$N_{neurons}$ is the number of neurons of the trained ANN
[28]	CNN-based completed distance refinement and DNN-based recovery scheme	$O\left(6U^{3}+9U^{2}\right)$	*U* is the total number of sensing nodes, including *M* known reference points (RPs) and *N* unknown points (UPs) to be localized
[24]	KNN-based and Naive Bayes-based methods	$O\left(mn\right)$	*m* is the number of possible transmitters to verify RSSI measurement; *n* is the number of comparisons performed between RPs and UPs on RSSI measurement
[130]	Local Gaussian Process method for fingerprint indoor localization based on WLAN radio map	$O\left(nL+L^{3}\right)$	*n* is the number of RPs; and *L* is the number of RPs in a training set
[37]	weight estimation of Unscented Kalman Filter (UKF)	$O\left(L^{2}\right)$	*L* is the number of weights
[57]	high dimensional state estimation by Cubature Kalman Filters (CKF)	$O\left(n^{3}\right)$	*n* is the number of state-vector dimensions
